# *De novo* design, retrosynthetic analysis and combinatorial synthesis of a hybrid antiviral (VTAR-01) to inhibit the interaction of SARS-CoV2 spike glycoprotein with human angiotensin-converting enzyme 2

**DOI:** 10.1242/bio.054056

**Published:** 2020-10-19

**Authors:** Vishvanath Tiwari

**Affiliations:** Department of Biochemistry, Central University of Rajasthan, Bandarsindri, Ajmer 305817, India

**Keywords:** Hybrid antiviral molecule, SARS-CoV2 spike glycoprotein, Angiotensin converting enzyme 2, *De novo* designing, Retrosynthetic analysis, Molecular dynamics simulation

## Abstract

SARS-like coronavirus (SARS-CoV2) has emerged as a global threat to humankind and is rapidly spreading. The infectivity, pathogenesis and infection of this virus are dependent on the interaction of SARS-CoV2 spike protein with human angiotensin converting enzyme 2 (hACE2). Spike protein contains a receptor-binding domain (RBD) that recognizes hACE-2. In the present study, we are reporting a *de novo* designed novel hybrid antiviral ‘VTAR-01’ molecule that binds at the interface of RBD-hACE2 interaction. A series of antiviral molecules were tested for binding at the interface of RBD-hACE2 interaction. *In silico* screening, molecular mechanics and molecular dynamics simulation (MDS) analysis suggest ribavirin, ascorbate, lopinavir and hydroxychloroquine have strong interaction at the RBD-hACE2 interface. These four molecules were used for *de novo* fragment-based antiviral design. *De novo* designing, docking and MDS analysis identified a ‘VTAR’ hybrid molecule that has better interaction with this interface than all of the antivirals used to design it. We have further used retrosynthetic analysis and combinatorial synthesis to design 100 variants of VTAR molecules. Retrosynthetic analysis and combinatorial synthesis, along with docking and MDS, identified that VTAR-01 interacts with the interface of the RBD-ACE2 complex. MDS analysis confirmed its interaction with the RBD-ACE2 interface by involving Glu35 and Lys353 of ACE2, as well as Gln493 and Ser494 of RBD. Interaction of spike protein with ACE2 is essential for pathogenesis and infection of this virus; hence, this *in silico* designed hybrid antiviral molecule (VTAR-01) that binds at the interface of RBD-hACE2 may be further developed to control the infection of SARS-CoV2.

## Abstract (precis)

**Summary:** SARS-CoV2 has caused an outbreak globally and is responsible for high mortality and morbidity. Interaction of the receptor-binding domain of spike protein of this virus with human angiotensin converting enzyme (ACE2) is vital for the infection. Hence, a *de novo* designed hybrid antiviral molecule (VTAR-01) targeting RBD-ACE2 interaction may play a very significant role in controlling the COVID-19 disease.

## INTRODUCTION

The emergence of novel coronavirus and the rapid outbreak of SARS-like coronavirus (SARS-CoV2) infection is endangering global health and the economy (Wu et al., 2020; Huang et al., 2020). On 11 March 2020, the World Health Organization (WHO) declared the outbreak a pandemic. By 7 August 2020 there were >1,90,00,000 cumulative cases globally and >7,00,000 deaths with ∼5% mortality rate in outcome cases. The main risk factors include older age, comorbidities, low lymphocyte count and high RALE (radiographical assessment of lung edema) (Ciceri et al., 2020). The average incubation period of this virus is 2 to 14 days (mainly 3 to 7 days), but it may be enhanced in people with histories of long-term use of glucocorticoids (Han et al., 2020). Additionally, there is a decrease in CD8^+^ T cells and NK cells in COVID-19 patients (Jiang et al., 2020) that is associated with worse prognosis and systemic inflammation (Urra et al., 2020).

Receptor recognition by coronavirus is a determinant for its infection, pathogenicity and human interaction (Perlman and Netland, 2009). Virus infection is initiated with the binding of viral particles to the host surface receptors. The receptor recognition by coronavirus involves the receptor-binding domain (RBD) of the spike protein of SARS-CoV2 (Shang et al., 2020) and human angiotensin-converting enzyme 2 (ACE2; Letko et al., 2020). It was reported that the ACE2 gene is hypomethylated and overexpressed in lupus T cells, and epigenetic dysregulation of ACE2 might suggest COVID-19 susceptibility and severity in lupus patients (Sawalha et al., 2020). As the receptor recognition is an important determinant for infection of coronavirus RBD of this protein contains a core and a receptor-binding motif (RBM) that mediates its contact with the human ACE2 (Shang et al., 2020). The hACE2 contains two virus-binding hotspots that are important for SARS-CoV2 binding (Wu et al., 2012). The recent study by Shang et al. (2020) has shown the residues Leu455, Phe486, Gln493, Ser495, Asn501 of RBD of SARS-CoV2, and Met82, Lys31, Glu35, Asp38, and Lys353 of ACE2 as important for the interaction of this virus with the human host. This shows that these residues can play a vital role in host–pathogen interaction and the pathogenesis of this virus. An inhibitor targeted at the interface of this interaction can play a significant role in preventing the pathogenicity of this virus.

Recently different non-specific antiviral and other therapeutics repurposing approaches were used to minimize the losses due to infection by SARS-CoV2, but their possible mechanism of action has not been investigated yet. Broad-spectrum antiviral molecules like remdesivir, ribavirin, lopinavir and hydroxychloroquine are currently used to treat COVID-19 (Singh et al., 2020). Hydroxychloroquine has been proposed to inhibit the virus entry, but its mechanism of action is unclear (Liu et al., 2020). Regulators have different opinions on the use of some of the recommended molecules (Jaffe, 2020). The glycation of spike protein is also important for the interaction (Walls et al., 2016) and spike protein of SARS-CoV2 and ACE2 have shown to be glycosylated (Wrapp et al., 2020; Zhou et al., 2020) and hence it is also essential to include glycans in studies to have a better picture of host–pathogen interaction.

There is no specific medicine available for the treatment of COVID-19; hence it is an urgent requirement of the current time to develop a novel specific antiviral that interferes in the interaction of SARS-CoV2 with the human host. In the present study, we have used *de novo* designing, retrosynthetic analysis, combinatorial synthesis and molecular dynamics simulations to design hybrid molecules that target the interaction of spike's RBD and hACE2 receptor. The designed antiviral VTAR-01 may inhibit the interaction of RBD of spike protein of SARS-CoV2 with hACE2.

## RESULTS

### Selection of interface of SARS-CoV2 spike protein and human ACE2 interaction

The PDB structure of the SARS-CoV2 RBD of spike protein complexed with ACE2 was taken from RCSB (PDB number 6VW1, 2.68 Å). This PDB structure consists of four subunits with two subunits (A and B) of ACE2 and two subunits (E and F) of RBD, metal ion (zinc, chloride) and carbohydrate moiety (beta-D-mannose, N-acetyl-glucosamine). The subunits A and B of ACE2, and E and F of RBD in the PDB structure are identical; hence one identical subunit (B and F) was removed from the PDB structure. The glycation is shown to be important for the interaction between spike protein and ACE2, hence beta-D-mannose and N-acetyl-glucosamine of A and E subunits of the complex have been considered for the present study. The RBD-ACE2 complex was prepared, optimised and minimised using OPLS-2005 force fields. The recent study by Shang et al. (2020) has shown that Leu455, Phe486, Gln493, Ser495, Asn501 of RBD of the spike protein of SARS-CoV2, and Met82, Lys31, Glu35, Asp38, and Lys353 of ACE2 are present at the interface of the interaction and play a significant role in the host–pathogen interaction. The present study aims to design an inhibitor that targets this interface of RBD and ACE2 interaction; hence we have used these amino acid residues for the grid generation for docking and considered for further studies.

### Selection of antiviral molecules

The antiviral molecules were selected based on the available literature search and their efficacy on the SARS-CoV2. The umifenovir and its analogs, lopinavir, ribavirin, hydroxychloroquine, ascorbate, remdesivir, oseltamivir, ritonavir and tamoxifen were selected for the present study. The ligand preparation resulted in 139 selected tautomer of these antiviral molecules that were used for its interaction with the RBD-ACE2 interface. The selection of novel antiviral molecules has been discussed in the coming sections.

### Docking at XP mode identified an antiviral molecule that binds at the interface of the RBD–ACE2 complex

**Fig. 1.**
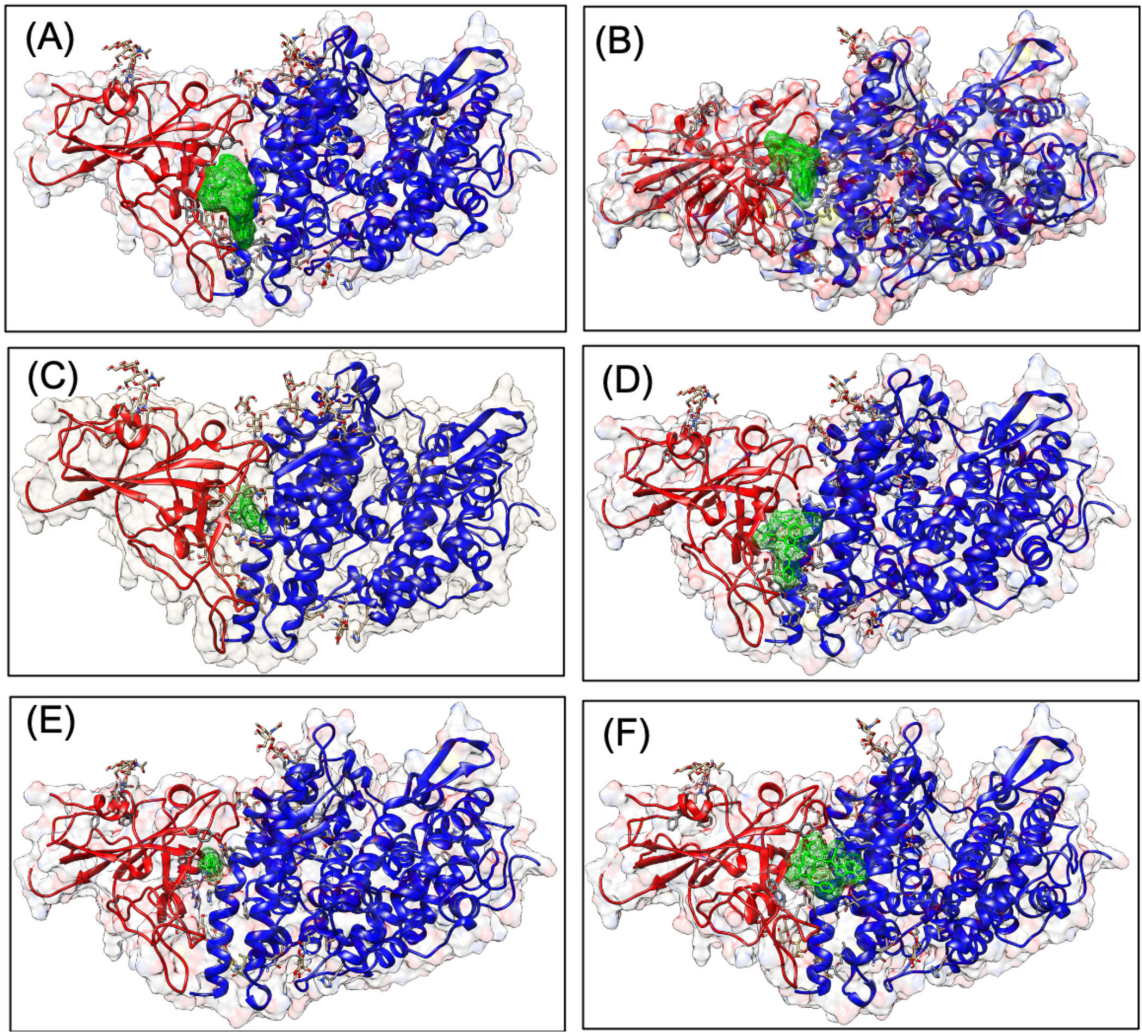
**Interaction diagram showing docking pose of lopinavir (A), hydroxychloroquine, ribavirin (C), umifenovir (D), ascorbate (E) and remdesivir (F) at the interface of the receptor-binding domain of SARS-CoV2 spike glycoprotein and human ACE2.** Docking grid used generated using binding residues at the interface of interaction.

We performed docking studies using Glide's Extra Precision XP mode. XP docking suggests that all the selected molecules docked with negative binding energy. Ascorbate, ribavirin, hydroxychloroquine, umifenovir, favipiravir and lopinavir have shown better interaction than other antiviral molecules studied. The docking poses are shown in Fig. 1, and docking scores, along with binding energies, are shown in Table 1. The interacting residues of the RBD-ACE2 interface that interact with individual antiviral molecules are shown in Table 2. It was observed that some molecules have better interaction with residues of RBD while others have better interaction with the human ACE2, but they all docked at the interface of the RBD-ACE2 complex.

**Table 1.**
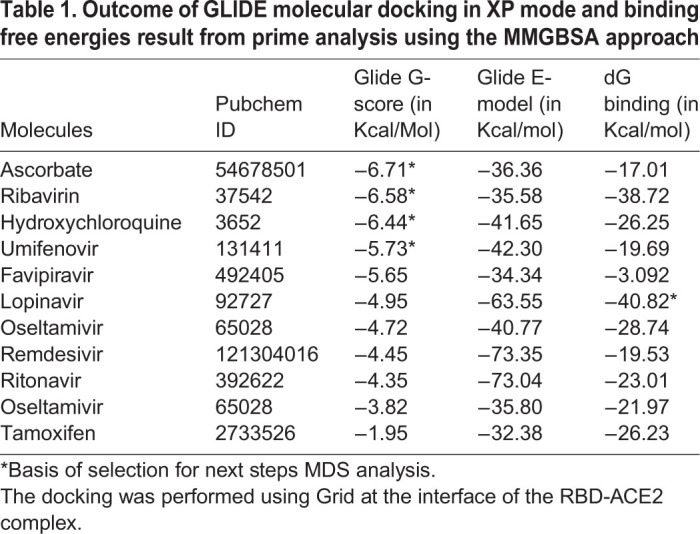
**Outcome of GLIDE molecular docking in XP mode and binding free energies result from prime analysis using the MMGBSA approach**

**Table 2.**
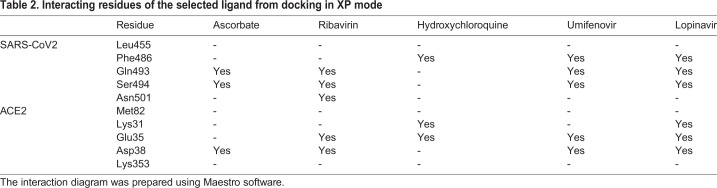
**Interacting residues of the selected ligand from docking in XP mode**

### Binding free energy calculations using molecular mechanics confirms the interaction of antiviral molecules with the RBD-ACE2 complex

All the antivirals underwent molecular mechanics analysis with generalised born and surface area solvation (MM-GBSA) methods to calculate the Gibbs free energy of binding. The result of this analysis is listed in Table 1. It was found that all the molecules have favourable Gibbs free energy change for binding to the RBD-ACE2 complex. Lopinavir has the best binding energy (−40.8 Kcal/mol), followed by ribavirin (−38.72 Kcal/mol). Based on docking and binding free energy results, ascorbate, ribavirin, hydroxychloroquine, umifenovir and lopinavir are selected for molecular dynamics analysis (MDS) and further study.

### MDS analysis confirms that ascorbate, ribavirin, lopinavir and hydroxychloroquine interact at the interface of the RBD-ACE2 complex

**Fig. 2.**
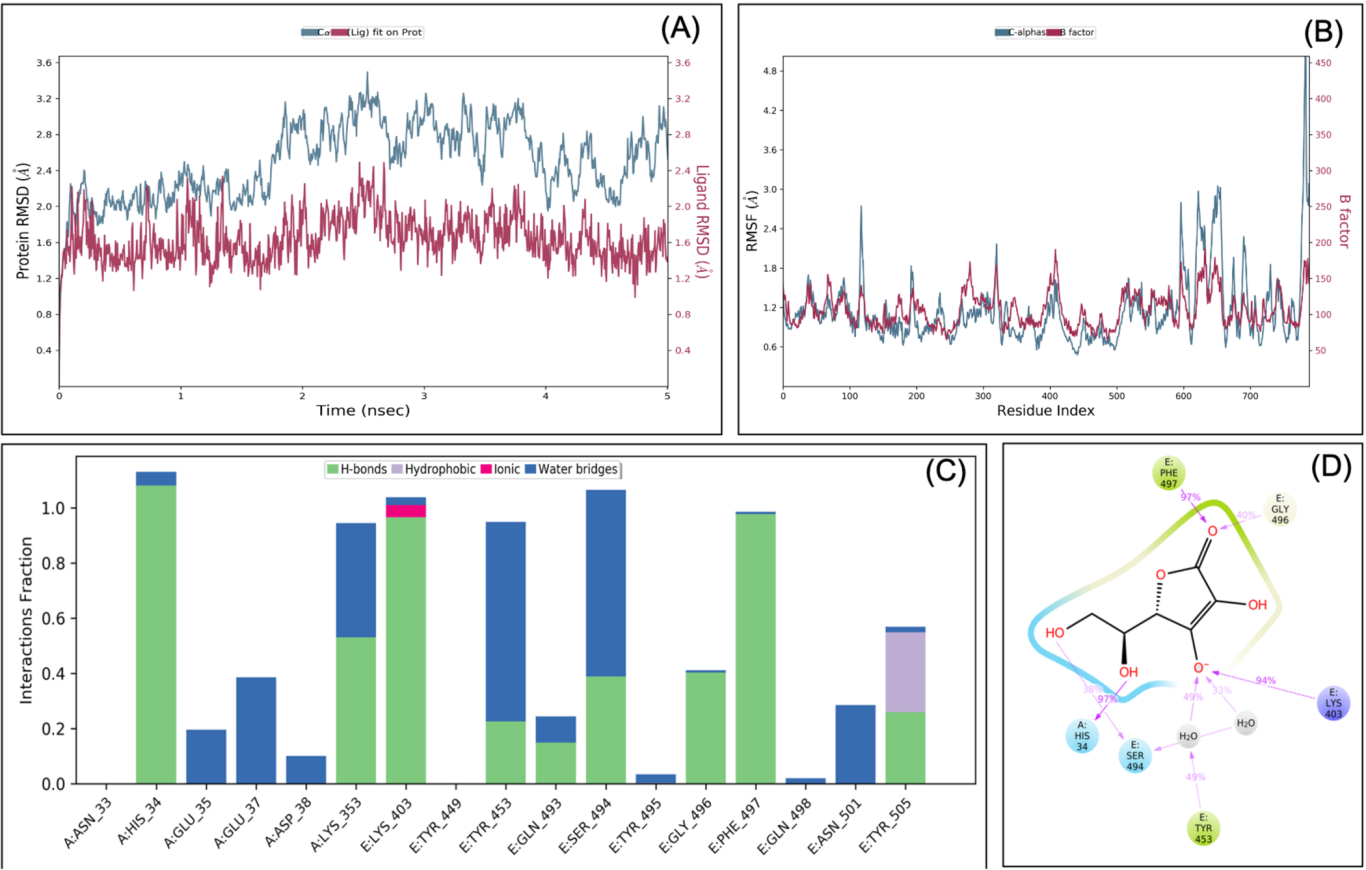
**Root-mean-square deviation (A) and root mean square fluctuations (B), interacting residues (C) and the interacting fraction (D) during molecular dynamics simulation analysis of the RBD-hACE2-ascorbate complex.**

MDS was performed for different ligand-protein complexes such as RBD-ACE2-ascorbate, RBD-ACE2-ribavirin, RBD-ACE2-lopinavir, RBD-ACE2-umifenovir and RBD-ACE2-hydroxychloroquine, up to 5 ns, and results were analysed for RMSD, RMSF, ligand stability and bonding. MDS analysis of the RBD-ACE2-ascorbate complex showed that RMSD of protein and ligands of <3 Å showed a stable complex (Fig. 2). RMSF study showed that the majority of the protein (except terminal) has the RMSF <2 Å, which showed less flocculation in protein conformation and hence a stable structure. Interaction between ascorbate and the RBD-ACE2 complex involves His34, Glu37, Lys353, Lys403, Tyr453, Ser494, Gly496, Phe497, Asn501 and Tyr505 and at least seven contacts always exist with more than 30% simulation time (Fig. 2D). It forms six hydrogen bonds in the same simulation time that showed that the interaction is specific.

**Fig. 3.**
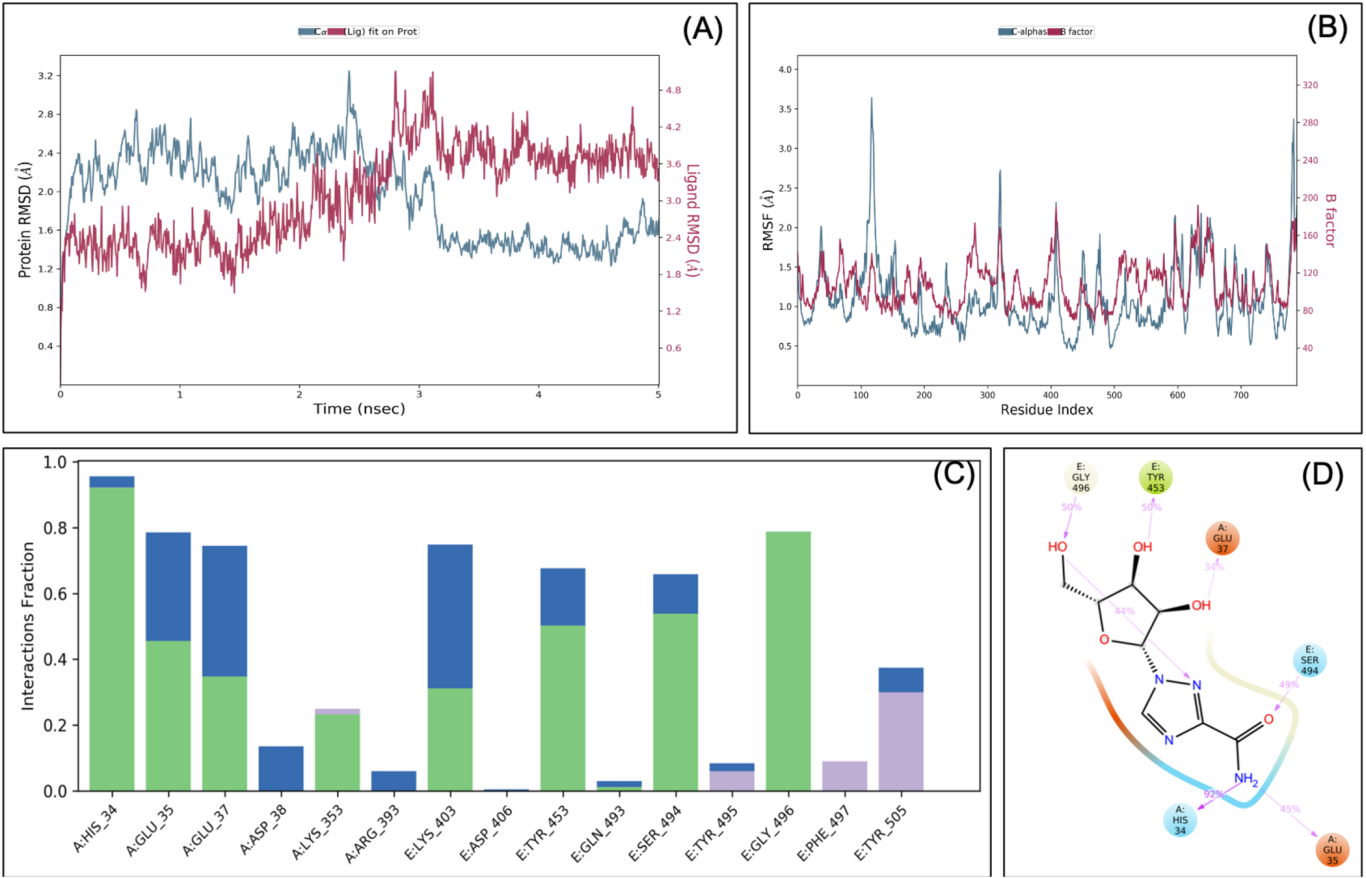
**Root-mean-square deviation (A), Root mean square fluctuations (B), Interacting residues (C), interacting fraction (D) during molecular dynamics simulation analysis of RBD-hACE2-ribavirin complex.**

Similarly, MDS analysis of the RBD-ACE2-ribavirin complex has RMSD <1.6 Å at 5 ns, which suggests a very stable complex (Fig. 3). RMSF study showed that the majority of the protein (except terminal) has the RMSF <2 Å, which showed a stable structure with some flocculation in protein conformation at the terminal. The amino acids involved in the interaction between ribavirin and the RBD-ACE2 complex are His34, Glu35, Glu37, Lys403, Tyr453, Ser494, Gly496 and Tyr505, and six contacts always exist between ribavirin and RBD-ACE2 in more than 30% of the simulation time (Fig. 3D). Similarly, MDS analysis of the RBD-ACE2-lopinavir complex showed that RMSD of protein and ligands have RMSD <2 Å at 5 ns that showed a very stable complex (Fig. S1). RMSF of this complex was found to be <2 Å that showed less flocculation in protein conformation, and hence a stable structure. Lopinavir interacts with the RBD-ACE2 complex via Lys31, Glu35, Leu79, Tyr449, Glu484, Tyr489, Phe490, with at least nine contacts that always exist in more than 30% of the simulation time (Fig. S1D). Although the strength of the interaction is good, it only forms three hydrogen bonds at the same time, which makes this interaction less specific.

Similarly, MDS analysis of the RBD-ACE2-umifenovir complex showed protein RMSD of <3 Å (Fig. S2), but ligand has an RMSD of <5 Å that showed its limited stability in the complex. RMSF analysis showed an RMSF <2 Å that showed less flocculation in protein conformation. Interaction between umifenovir and the RBD-ACE2 complex involves Lys31, Glu35, Lys68, Phe72 and Tyr489, and at least four contacts always exist in more than 30% of the simulation time (Fig. S2D), with one hydrogen bond. This showed that this complex is relatively less stable than other complexes. In addition, MDS analysis of the RBD-ACE2-hydroxychloroquine complex showed that RMSD of protein has an RMSD of <3 Å that showed a stable complex (Fig. S3), but ligand has RMSD of <15 Å, which showed its limited stability in the complex. RMSF analysis showed that the majority of the protein (except terminal) has the RMSF <2 Å that showed less flocculation in protein conformation, and hence a stable protein structure. Interaction between hydroxychloroquine and the RBD-ACE2 complex involves Glu75 and Glu484, and no interaction exists in more than 30% of the simulation time (Fig. S3D). This showed that this complex is relatively less stable than other complexes investigated.

Comparative interacting residues analysis in the MDS results showed that ascorbate and ribavirin have the best interaction and interact with both RBD and ACE2. Similarly, lopinavir has a strong interaction with ACE2. In addition, hydroxychloroquine forms a very strong interaction with Glu484 residue of RBD, which is in the binding pocket but not involved in physical interaction with ACE2. Hence, from MDS result analysis, ascorbate, ribavirin, lopinavir and hydroxychloroquine were selected for *de novo* fragment-based drug design.

### *De novo* fragment-based designing and MDS analysis identified VT-AR as a hybrid antiviral targeted at the RBD-ACE2 interface

**Fig. 4.**
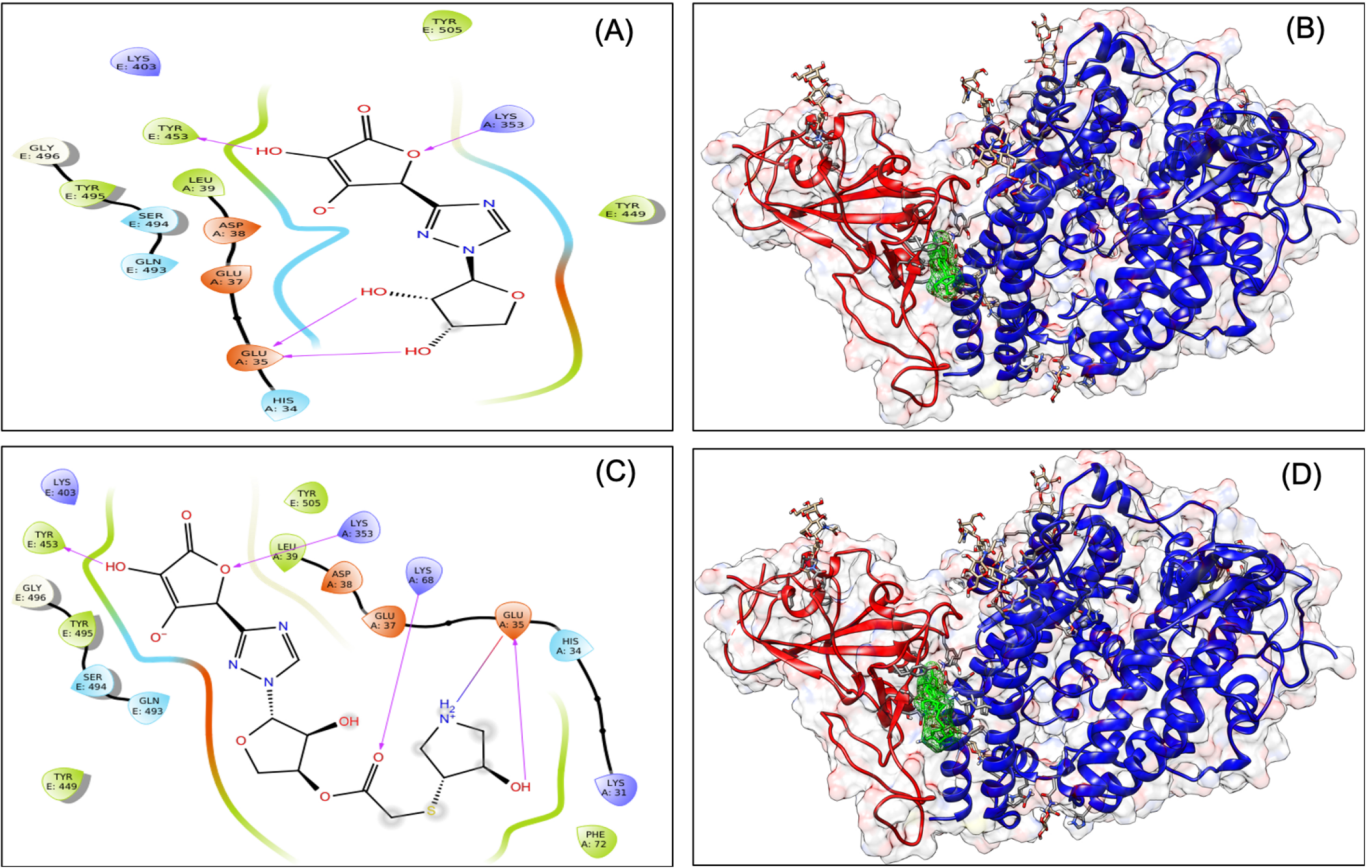
**Interaction diagram showing interacting amino acid residues and their docking pose in RBD-hACE2-VTAR complex (A and B), RBD-hACE2-VTAR-01 complex (C and D).**

**Fig. 5.**
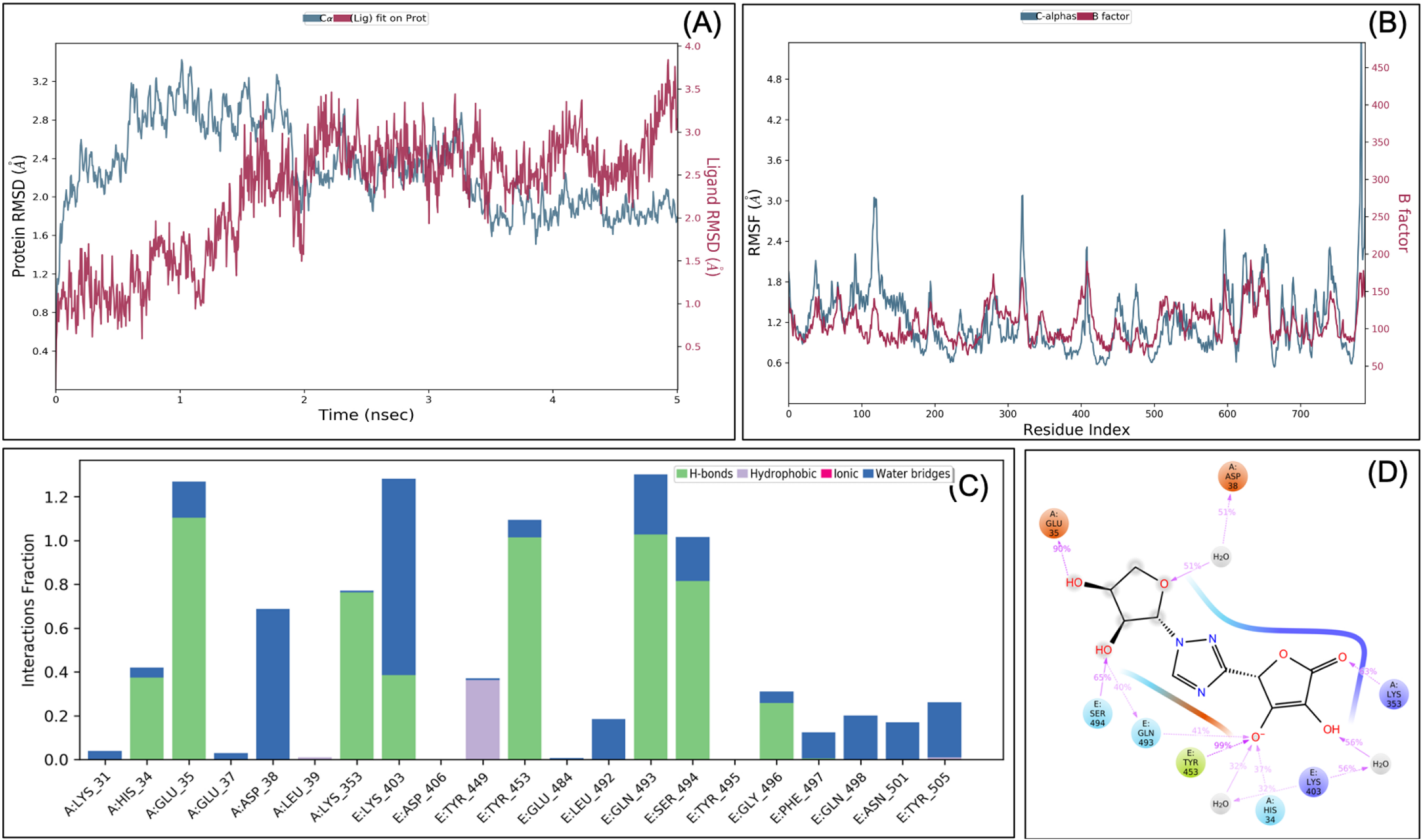
**Root-mean-square deviation (A), root mean square fluctuations (B), interacting residues (C), and interacting fraction (D) during molecular dynamics simulation analysis of RBD-hACE2-VTAR complex.**

Denovo fragment-based drug designing was started with the fragmentation of the selected molecules, i.e. ascorbate, ribavirin, lopinavir, and hydroxychloroquine. A total of 414 fragments were produced that includes 386 fragments of lopinavir, 21 fragments of hydroxychloroquine, five fragments of ribavirin, and two fragments of ascorbate. All the 414 fragments were docked (in SP mode) to the grid of the RBD-ACE2 complex. On the basis of a manual analysis of docked complexes, fragments 1 and 4 of ribavirin, fragments 1 and 2 of ascorbate, fragments 6, 8, 15, 17, 21 of hydroxychloroquine and fragment 122, 214, 350, 361, 373 of the lopinavir were selected for *de novo* fragment-based design. This produces 14 combinations, which along with four original molecules, were prepared via LigPrep that produces a total of 139 tautomers. All 139 tautomers underwent virtual screening for their binding to the grid at the RBD-ACE2 interface. The two fragments (a hybrid of fragment 4 of ribavirin and fragment 1 of the ascorbate) showed the best docking. Their docking score (−7.04) was found to be higher than their parent molecules (Table S1) and named as ‘VTAR’, and chemically it is (2S)-2-{1-[(2S,3S,4S)-3,4-dihydroxyoxolan-2-yl]-1H-1,2,4-triazol-3-yl}-4-hydroxy-5-oxo-2,5-dihydrofuran-3-olate (Fig. 4A and B). To further confirm the interaction, the MDS analysis of the RBD-ACE2-VTAR complex was performed that showed that RMSD of both protein and ligands are <2 Å that showed stable complex (Fig. 5). RMSF analysis showed that the most of the protein (except terminal) has the RMSF <3 Å that showed stable complex some flocculation in protein conformation at terminals. Interaction between VT-AR and RBD-ACE2 complex involves His34, Glu35, Asp38, Lys353, Lys403, Tyr 449, Tyr453, Gln493, Ser494 and Gly496, and at least ten contacts always exist with more than 30% simulation time (Fig. 5D) and eight hydrogen bonds between VTAR and RBD-ACE2 complex, which showed a very strong and specific interaction.

### Retrosynthetic analysis, combinatorial synthesis and MDS analysis identified VTAR-01 as a hybrid antiviral targeted to the RBD-ACE2 interface

**Fig. 6.**
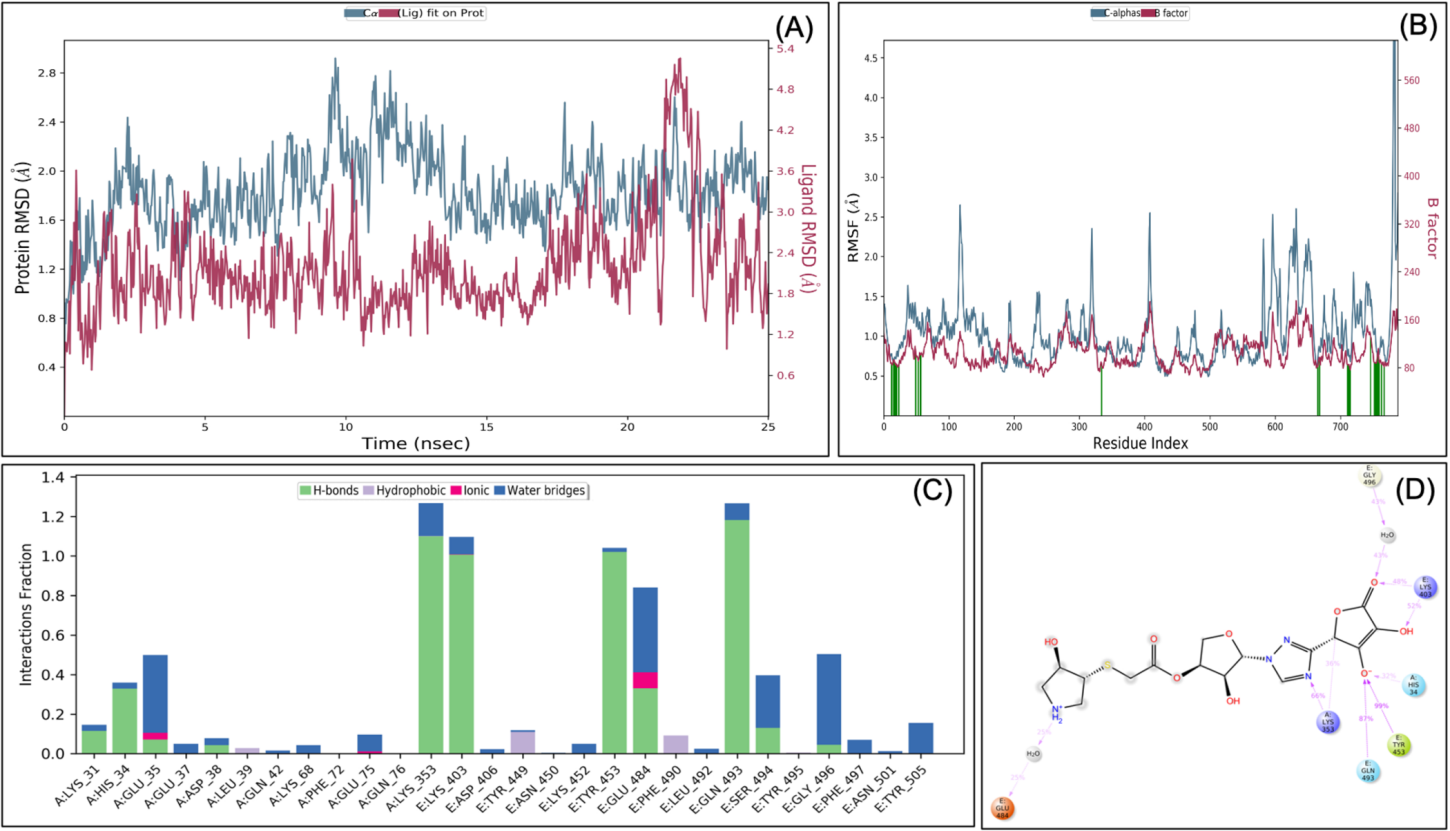
**Root-mean-square deviation (A), root mean square fluctuations (B), interacting residues (C), and interacting fraction (D) during molecular dynamics simulation analysis of RBD-hACE2-VTAR01 complex.** Experimental was performed until 25 ns, and 1000 frames were recorded throughout the simulation.

To produce synthetically tractable lead-like compounds, retrosynthetic analysis and combinatorial synthesis was performed, which produces 100 products of VTAR with more than 100 modifications. The 100 products were prepared and docked to the RBD-ACE2 complex. Docking and binding energy calculation identified VTAR-01 as best docked molecule (-7.8) and identified as (3R,4R)-3-hydroxy-4-[(2-{[(3S,4S,5S)-4-hydroxy-5-{3-[(2S)-4-hydroxy-3-oxido-5-oxo-2,5-dihydrofuran-2-yl]-1H-1,2,4-triazol-1-yl}oxolan-3-yl]oxy}-2-oxoethyl) sulfanyl] pyrrolidin-1-ium (Fig. 4C and D). To further confirm the interaction, the MDS analysis of the RBD-ACE2-VTAR-01 complex was performed up to 25 ns, and the result showed that RMSD of both protein and ligands are <2.5 Å that showed a very stable complex (Fig. 6). RMSF analysis showed that the most of the protein (except terminal) have the RMSF <3 Å at most of the simulation time that showed less flocculation in protein conformation. Interaction between VTAR-01 and RBD-ACE2 complex involves His34, Glu35, Lys353, Lys403, Tyr453, Glu484, Gln493, Ser494 and Gly496, and at least nine contacts always exist more than 30% simulation time (Fig. 6D). The comparative analysis showed that VTAR-01 forms eight hydrogen bonds with the RBD-ACE2 complex, which shows specific binding with VTAR-01. Comparative analysis of all the results showed that VTAR-01 has the highest docking score (-7.8), has eight hydrogen bonds, and interacts with Glu35 and Lys353 of ACE2, as well as Gln493 and Ser494 of RBD, confirming its strong interaction with the RBD-ACE2 interface. It is also noted in MDS analysis that interaction of VTAR-01 with the Glu35 and Gln493 exists at 100% simulation time. This further confirms its strong interactions at the interface of the RBD-ACE2 complex.

### Designed VTAR-01 has favourable ADMET properties

Absorption, distribution, metabolism, excretion and toxicity (ADMET) analysis of VTAR-01 was performed, and the result showed that most of its ADMET parameters are within the acceptable limits (Table S2) that further support the designed leads. QPlogPw, QPlogPo/w, QPlogS showed that VTAR-01 is water-soluble. The availability of free drugs in the blood is essential for them to cross the membrane and bind to molecular targets. The QPlogKhasa showed that the VTAR-01 has very low interaction with plasma proteins.

### Designed VTAR-01 has no human off-target effects and no predicted cytotoxicity in the cell line

The presence of lead's off-target effects in humans may reduce efficacy and cause cytotoxicity. Hence, human off-targets and cytotoxicity of VTAR-01 in cell lines were predicted. The result showed no human off-targets of this VTAR-01. Similarly, the predicted cytotoxicity of the VTAR-01 has been demonstrated that it is non-cytotoxic to tumour and non-tumour cell lines (at Pa>0.6). This further enhances the possibility of the development of VTAR-01 as a possible lead.

## DISCUSSION

Infection caused by SARS-CoV2 has emerged as a global pandemic. The spike protein of coronavirus facilitates viral entry into target cells. Human receptor recognition by this protein is a vital infection determinant for coronavirus. The receptor recognition by coronavirus involves the RBD of the spike protein of SARS-CoV2 (Shang et al., 2020) and hACE2 (Letko et al., 2020). The recent study, Shang et al. (2020), showed that Leu455, Phe486, Gln493, Ser495, Asn501 of RBD of spike protein, and Met82, Lys31, Glu35, Asp38 and Lys353 of ACE2 directly interact with each other. Currently, no specific FDA-approved treatment is available for the infection caused by SARS-CoV2. There are different non-specific treatments for COVID-19 that are presently used, such as remdesivir, ribavirin, lopinavir and hydroxychloroquine (Singh et al., 2020), but their mechanism of action on SARS-CoV2 has not been investigated to date. Ribavirin is a guanosine analog that is reported to have multiple mechanisms of action (Loustaud-Ratti et al., 2016) and has also been shown to have an inhibitory effect against SARS-CoV2 (Khalili et al., 2020). Lopinavir, an HIV type 1 Aspartate protease inhibitor, has activity against other coronaviruses like SARS-CoV (Chu et al., 2004). It is reported that lopinavir-ritonavir with ribavirin and interferon alfa resulted in a patient's survival of MERS-CoV infection (Kim et al., 2016). Remdesivir (GS-5734) is currently used with lopinavir against COVID-19 without knowing its targets in SARS-CoV (Choy et al., 2020). Ritonavir, lopinavir and remdesivir have been used to control other coronaviruses (Sheahan et al., 2020). Recent clinical trials have shown that the broad-spectrum antivirals lopinavir and ritonavir alone were ineffective against COVID-19 (Cao et al., 2020). Recently, hydroxychloroquine has been found to have activity against SARS-CoV2 (Gao et al., 2020), and it was found to be more effective than chloroquine (Yao et al., 2020). Recent reports also suggest that hydroxychloroquine did not prevent illness when used as post-exposure prophylaxis within 4 days after exposure (Boulware et al., 2020). Ascorbate has shown a positive effect on another SARS coronavirus, and it has shown multiple mechanisms of action (Hemilä, 2003). In the present study, the interaction between RBD of the spike protein of SARS-CoV2 and human ACE2 is targeted by novel hybrid antiviral VTAR-1, which was designed using *de novo* designing, retrosynthetic analysis, combinatorial synthesis and molecular dynamic simulation.

PDB structure of the RBD-ACE2 complex was retrieved from the RCSB, and the grid was prepared at the interface of this interaction. The docking, binding energy calculation and the molecular dynamic simulation calculation have shown that ascorbate, ribavirin, hydroxychloroquine, umifenovir and lopinavir have good interactions with this interface. It has been shown that ascorbate has some preventive effect in infection by the other coronaviruses (Hemilä, 2003), but its preventive role in the infection of SARS-CoV2 is under Phase II clinical trial (clinicaltrails.gov identifier: NCT04264533) and its outcome is expected by September 2020. The present study highlighted the molecular mechanism of the ascorbate (Vitamin C) to prevent the infection by SARS-CoV2. In addition to that, this study also explains the probable molecular mechanism of hydroxychloroquine in preventing the infection by SARS-CoV2. Experimental studies have shown that hydroxychloroquine is effective against SARS-CoV2 (Yao et al., 2020), but its mechanism of action is not studied. Various *in vitro* experimental data suggest that the ribavirin has shown an inhibitory effect on SARS-CoV2 (Khalili et al., 2020), but a possible mechanism of action is not clear to date. The present study also gives the molecular explanation for some success of ribavirin, ascorbate and lopinavir against the SARS-CoV2, but these broad-spectrum antiviral molecules are not specific to SARS-CoV2. Therefore, in the present study, we are reporting a specific hybrid inhibitor VTAR-01 targeted at the interaction point of the receptor-binding domain of spike protein with hACE2. It is also seen that the expression of the ACE2 receptor is not limited to the lungs, but its presence is also involved in extrapulmonary spread (Gu et al., 2005), which further enhances the importance of this study.

To find a novel inhibitor, we have used *de novo* fragment-based drug design using FDA-approved antiviral molecules like ascorbate, ribavirin, hydroxychloroquine and lopinavir, which showed good interaction with the RBD-ACE2 complex. A total of 414 fragments were generated from these molecules. They were docked to the interface of the RBD-ACE2 complex, and best-docked fragments were manually analysed of its interaction at the interface of this complex. The selected fragments were joined via *de novo* fragment-based drug design. This approach joined the different fragments based on the structural similarity to the active molecules and their scaffolds, hence used to navigate a huge chemical space (Kawai et al., 2014). The hybrid molecules were further confirming its interaction at the interface of RBD-ACE2 complex using the XP mode of docking and molecular dynamics simulations. This select VTAR as a possible *de novo* synthesized hybrid molecule that can interact at the interface via Gln493 and Ser 494 of RBD of SARS-CoV2 and Glu35, Asp38 and Lys 353 of hACE2.

Lead optimization required the design, synthesis and profiling of thousands of leads analogs prior to clinical candidate nomination that takes a longer time. Further, to explore possible improvement in the VTAR, we have used *in silico* based retrosynthetic analysis, combinatorial synthesis and MDS analysis to design a new hybrid molecule. Retrosynthetic analysis followed by combinatorial synthesis generated 100 analogs of the *de novo* designed hybrid molecules (VTAR) in synthetically accessible chemical space and investigated them for interaction with the RBD-ACE2 complex. All the analysis identified VTAR-01 as a better molecule than VTAR and has shown the best interaction at the interface of the RBD-ACE2 complex. MDS analysis confirms the interaction of VTAR-01 at the interface of the RBD-hACE2 complex via Gln493 and Ser 494 of RBD of SARS-CoV2 and Glu35 and Lys 353 of hACE2. This showed the modification in VTAR-01 by retrosynthetic analysis and combinatorial synthesis further enhance the docking and binding energy without changing its interacting positions at the interface of the RBD-hACE2 complex.

In addition to the inhibition of virus entry, it is also essential to investigate the other complications of SARS-CoV2 infection. During infection, there is a burst of massive inflammation in the lungs and other organs including the kidneys, and the cardiovascular and neurological systems, which is associated with cytokine release syndrome known as ‘cytokine storm’. The blockage of this cytokine storm is also vital along with inhibition of virus entry to other cells. Different approaches are investigated, such as blocking cytokine storm by rapamycin (Omarjee et al., 2020), and blood purification therapy (Ma et al., 2020). Similarly, blockage of IL-6 and granulocyte macrophage-colony stimulating factor inhibits monocyte-macrophage recruitment and differentiation to the lungs, and blocks the inflammatory response (Gómez-Rial and Martinón-Torres, 2020). In addition to that, deposition of immune complexes (ICs) inside vascular walls can also induce cytokine storm (Roncati et al., 2020) hence status of ICs may be too critical for design and immune-based treatments like plasma therapy and vaccine (Vuitton et al., 2020). To make the situation more complicated, there is an activation of complement C3 during lung injury, and C3-targeted intervention by compstatin-based complement C3 inhibitor AMY-101 prevents complement-mediated inflammatory damage in COVID-19 patients (Mastaglio et al., 2020). Antibody-based therapies also represent valuable treatment approaches to treat symptomatic patients and prophylactically in at-risk individuals (Yager, 2020). One such vaccine, i.e. recombinants adenovirus type-5 (Ad5) vectored COVID-19 vaccine, is tolerable and immunogenic at 28 days post-vaccination (Zhu et al., 2020). Presence of different antigens in connective tissue, cardiovascular, gastrointestinal and nervous systems that have cross-reaction with SARS-CoV2 antibodies further complicate the condition and cause autoimmunity (Vojdani and Kharrazian, 2020). Therefore, along with the inhibitor that blocks the interaction between spike protein with ACE2, the other factors like cytokine storm and autoimmunity may also be considered. A combination of different approaches may be suitable to control COVID-19.

Therefore, the present study concludes that *de novo* designing, retrosynthetic analysis and combinatorial synthesis, in a molecular dynamic simulation study, designed a novel, hybrid antiviral VTAR-1 that can bind at the interface of RBD of the spike protein of SARS-CoV2 with hACE2 receptor. This molecule VTAR-01 needs to be synthesised and experimentally tested against SARS-CoV2 in animals and humans before therapeutic use.

## MATERIALS AND METHODS

### Retrieval of the structure of SARS-CoV2 complexed with ACE2

The PDB structure of the SARS-CoV2 RBD of spike protein with ACE2 is available hence retrieved from RCSB (PDB number 6VW1, resolution 2.68 Å). This PDB structure also has zinc ion, chloride ion, ethylene glycol, beta-D-mannose, N-acetyl-glucosamine in A chain, and beta-D-mannose, N-acetyl-glucosamine in E-chain. This PBD is a tetramer (ACE2, A, and B; RBD, E, and F) with two identical sets. The B and F subunit was removed using Maestro. The selected RBD-ACE2 complex (A and E unit) was pre-processed by assigning bond orders, adding hydrogens, creating zero-order bonds for metals, creating disulphide bonds, minimised optimised for water orientation and pH, and minimised for converge heavy atoms to 0.3RMSD using OPLS_2005 force field. We identified the interacting residues of RBD and ACE2 as per Shang et al. (2020) and used for receptor grid generation Leu455, Phe486, Gln493, Ser495 and Asn501 of RBD, and Met82, Lys31, Glu35, Asp38 and Lys353 of ACE2 as binding residues. This receptor grid was used to screen a number of the antiviral molecules that targeted the interface of the interaction of RBD and ACE2.

### Ligand preparation

The antiviral currently prescribed for COVID-19, as well as targeting the virus membrane fusion, was selected in the present study. The selected antiviral drugs were umifenovir, and its analogs, lopinavir, ribavirin, hydroxychloroquine, ascorbate, remdesivir, oseltamivir, ritonavir and tamoxifen. The SDF structure of the antiviral molecules was downloaded from the PubChem database. These SDF structures, along with *de novo* designed hybrid antiviral molecules, were prepared by using LigPrep modules of the Schrödinger suite as per published protocol (Tiwari et al., 2018b).

### Docking of antiviral on the interface of the RBD-ACE-2 complex

All the prepared ligands were docked to the docking grid of RBD-ACE2 complex in extra precision (XP) mode as per published protocol (Verma and Tiwari, 2018).

### MM-GBSA calculations of selected library compounds

To get more accurate interaction, XP docked antiviral molecule with RBD-ACE2 complexes were further subjected to MM-GBSA calculations using the Prime module of the Schrödinger suite as per published methods (Tiwari et al., 2019).

### MDS analysis

MDS was performed using Desmond modules of the Schrödinger 2019–4 suite as per published methods (Wright et al., 2020). The protein-ligand complex was prepared and subjected to the system builder using the OPLS3e force field. The system was built for the protein-ligand complex using the TIP3P solvent model; sodium ion was added to make charge-neutral, 0.15 M NaCl was added to make the system close to the natural system. The simulation was run for 5 ns or 25 ns (VTAR01-protein complex), with 5 ps trajectory recording intervals. System energy was set to be 1.2, and the ensemble class used was NPT. The simulation was set to run at 300k at 1.01325 bar. The option to relax the system before simulation was selected. The simulated system was analysed for the simulation interaction diagram.

### *De novo* fragment-based drug design

*De novo* fragment-based drug designing is a newly emerged approach (Kawai et al., 2014). The different fragments of selected antiviral molecules were generated. The different fragments of ligands were docked into the binding site of the RBD-ACE 2 complex. The docked complex was manually analysed for its position of docking in the binding site. Different fragments (fragment 1 and 4 of ribavirin; fragment 122, 214, 350, 361 and 373 of lopinavir; fragment 1 of Ascorbate; fragment 6, 8, 15, 17, 21 of hydroxychloroquine) were selected based on their binding to the binding interface of the RBD-ACE2 complex. The selected fragments were joined using the Breed module to generate newly designed hybrid antiviral molecules. The designed antiviral molecules were further confirmed for their interaction with the binding interface of the RBD-ACE2 complex using XP-docking and molecular dynamics simulations analysis.

### Retrosynthetic analysis and combinatorial synthesis

To produce synthetically tractable lead-like compounds, retrosynthetic analysis, and combinatorial synthesis were performed for the *de novo* synthesised ‘VTAR’ hybrid antiviral molecule as per published method using PathFinder (Konze et al., 2019). *In silico* enumeration provides a more exhaustive exploration of available chemical space than traditional empirical SAR studies. PathFinder can incorporate more than 100 reactions like C-C bond formation, alkylation, ether formation, amide coupling and heterocyclic systems like imidazole, triazoles, oxazole, indole and pyridine that are required for the molecular scaffolds and drug discovery (Bemis and Murcko, 1996). A total number of 100 pathways were investigated to produce 100 products. The top 10% of product that is similar to the input molecule VTAR were selected for further analysis. The selected 100 products were analysed by docking and molecular dynamics simulations.

### ADMET analysis

ADMET analysis was carried out for the selected lead VTAR-01 using QikProp analysis as per published protocol (Tiwari et al., 2020).

### Identification of human off-targets of designed lead

The human off-targets of the designed lead were predicted using Swiss Target Prediction (Daina et al., 2019) using the published protocol (Tiwari et al., 2018a).

### Prediction of cytotoxicity of designed lead on cell lines

*In silico* cytotoxicity screening was performed by cell line cytotoxicity predictor (CLC-Pred) as per published protocol (Lagunin et al., 2018).

## Supplementary Material

Supplementary information
