# Supplementary material for: De novo design, retrosynthetic analysis and combinatorial synthesis of a hybrid antiviral (VTAR-01) to inhibit the interaction of SARS-CoV2 spike glycoprotein with human angiotensin-converting enzyme 2
Source: Biol Open. 2020 Oct 19;9(10):bio054056. doi: 10.1242/bio.054056 (PMC7595696; doi:10.1242/bio.054056)
Supplement: Supplementary information [file biolopen-9-054056-s1.pdf]

Supplementary Data

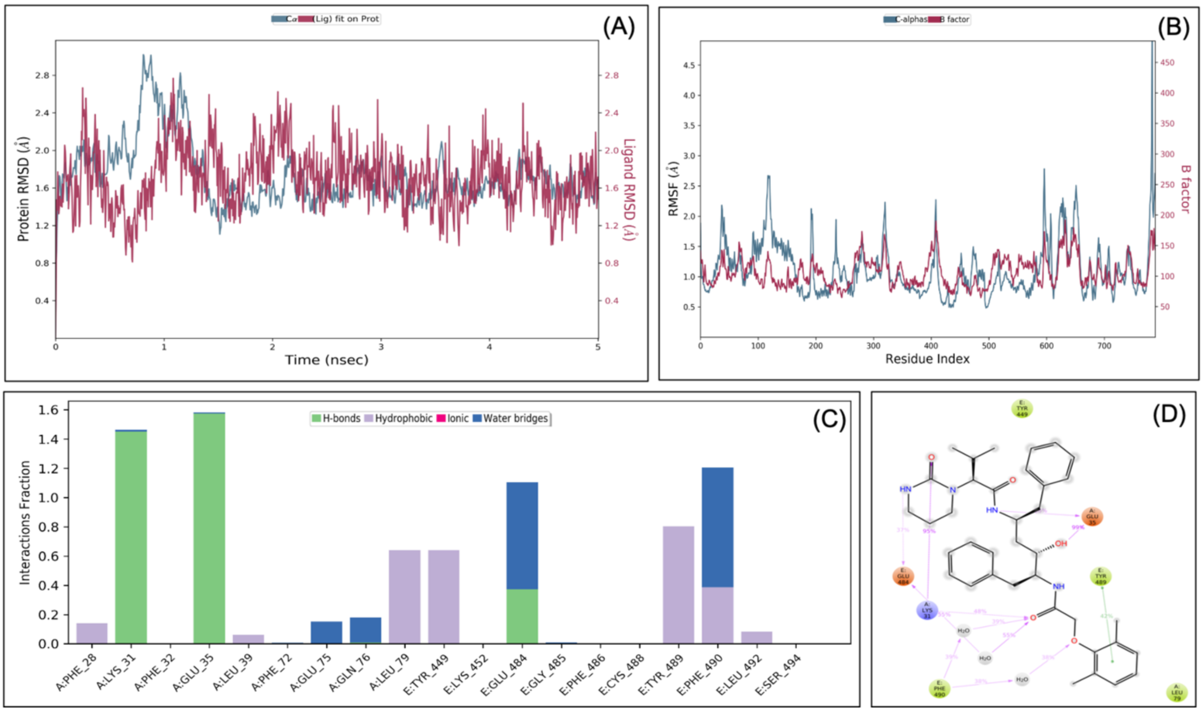

Figure S1: Root-mean-square deviation (A) and Root mean square fluctuations (B), Interacting residues (C), and the interacting fraction (D) during molecular dynamics simulation analysis of RBD-hACE2-lopinavir complex.

Biology Open • Supplementary information

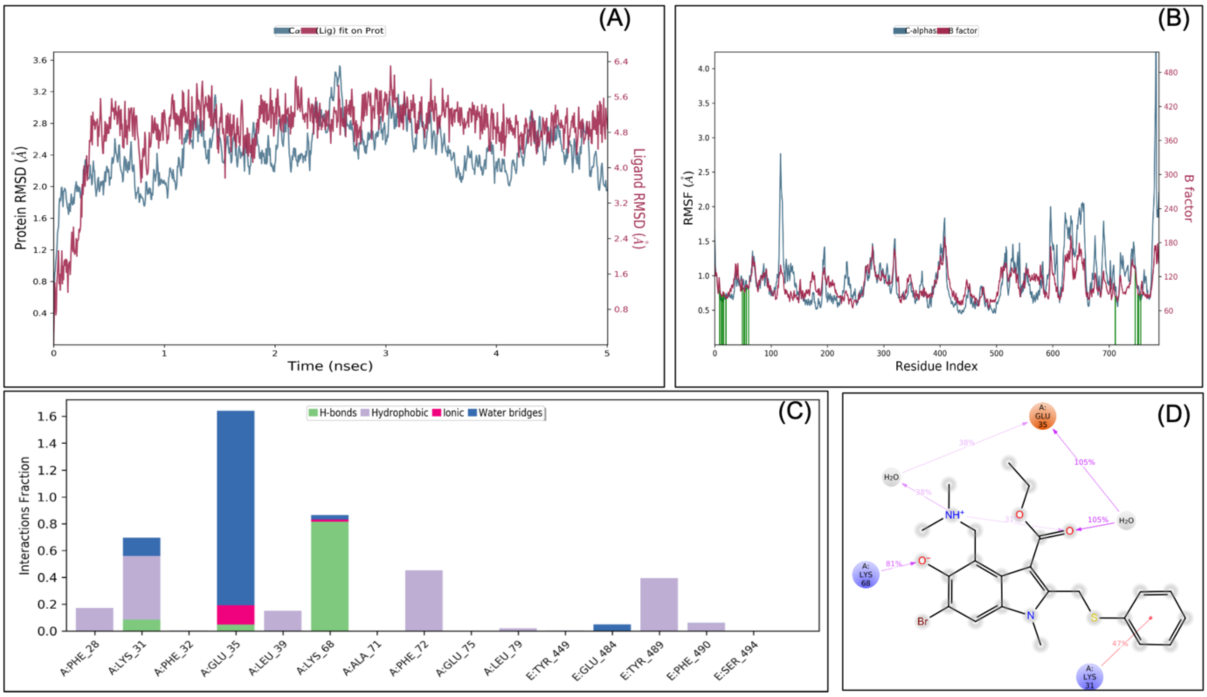

Figure S2: Root-mean-square deviation (A) and Root mean square fluctuations (B), Interacting residues (C), and the interacting fraction (D) during molecular dynamics simulation analysis of RBD-hACE2- umifenovir complex.

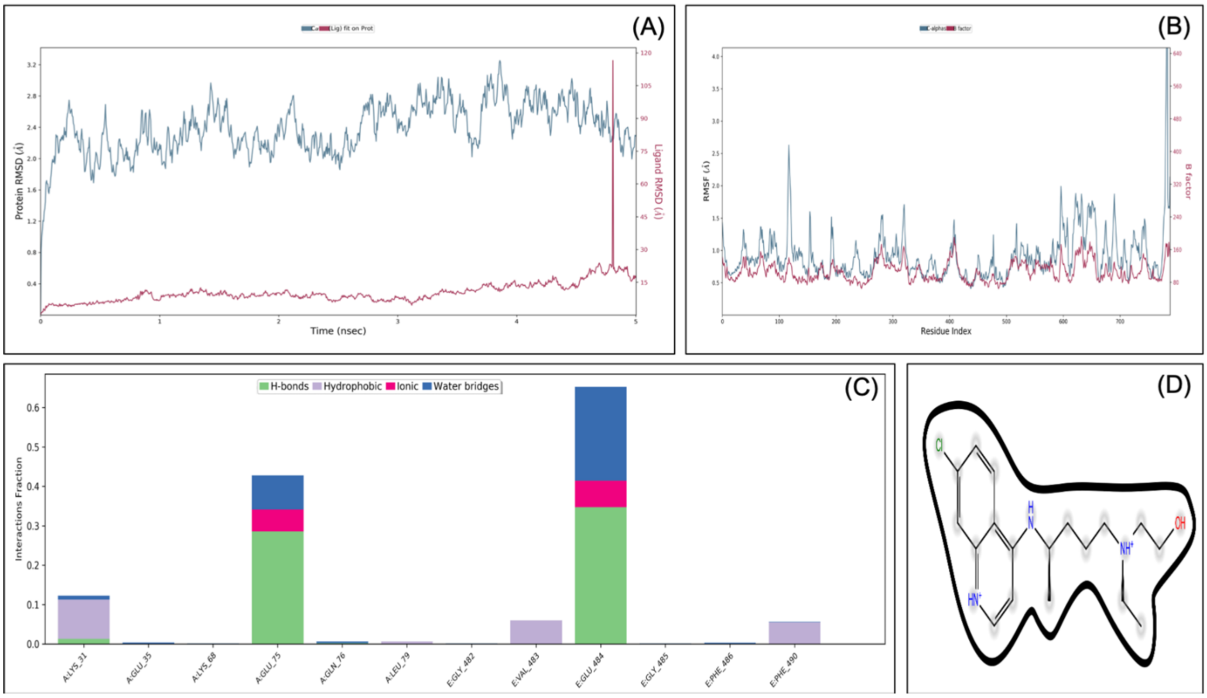

Figure S3: Root-mean-square deviation (A) and Root mean square fluctuations (B), Interacting residues (C) and the interacting fraction, (D) during molecular dynamics simulation analysis of RBD-hACE2-hydroxychloroquine complex.

Biology Open • Supplementary information

Table S1: Result showing outcome of GLIDE molecular docking in XP mode and Binding free energies result from Prime analysis using MMGBSA approach for the denovo designed hybrid molecule 'VTAR' and retrosynthesised hybrid molecule 'VTAR-01'

Name (Chemical Name)	Glide G-Score (in Kcal/Mol)	Glide E-Model (in Kcal/mol)	MMGBSA dG Binding (in Kcal/mol)
VT-AR<br><br>(2S)-2-{1-[(2S,3S,4S)-3,4-dihydroxyoxolan-2-yl]-1H-1,2,4-triazol-3-yl}-4-hydroxy-5-oxo-2,5-dihydrofuran-3-olate	-7.04	-45.56	-21.19
VT-AR-01<br><br>(3R,4R)-3-hydroxy-4-[(2-{[(3S,4S,5S)-4-hydroxy-5-{3-[(2S)-4-hydroxy-3-oxido-5-oxo-2,5-dihydrofuran-2-yl]-1H-1,2,4-triazol-1-yl}oxolan-3-yl]oxy}-2-oxoethyl)sulfanyl]pyrrolidin-1-ium	-7.80	-66.22	-29.00

Table S2: Result showing outcome of ADMET (Absorption, Distribution, Metabolism, Excretion and Toxicity)analysis of VT-AR-01. The prediction was performed using QikProp analysis.

ADMAT Properties	Value for VT-AR-01
Molecular Weight	444.415
H-bond Donor	5.0
H-bond Acceptor	16.6
Rotatable bonds	10
QP polrz	38.773
QPlogP16	13.973
QPlogPoct	30.714
QPlogPw	26.114
QPlogPo/w	-2.676
QPlogS	-1.691
QPlogHERG	-5.844
QPlogKhasa	-1.197
Metab	7

Biology Open • Supplementary information
